# Development of a Model System for Tasting Grain Varieties †

**DOI:** 10.3390/foods9040510

**Published:** 2020-04-17

**Authors:** Thao Tran, Edgar Chambers

**Affiliations:** Center for Sensory Analysis and Consumer Behavior, Kansas State University, Manhattan, KS 66506, USA; thaotran@ksu.edu.ECIV

**Keywords:** grain, lexicon, sorghum, model system, cookie, cooked, sensory

## Abstract

This study investigated suitable approaches and effective applications for the evaluation of grain flavor differences among cultivars. A model system that helps to facilitate the characterization of flavors in grain varieties was developed using sorghum grain as a tool. Five different applications were initially used, including cooked grain, porridge, cookies, muffins, and extruded puffed snacks. Six highly trained sensory panelists participated in the project. The effectiveness of each application was determined based on the results of the attribute generation process and from panelist feedback. The results indicate that the combination of a cooked whole grain procedure and the use of flour made into cookies provides an effective and potent model for flavor characterization in both their grain form and as finished products. Both the recipes for the cooked grain and cookie applications effectively brought out the flavor characteristics of the grains as well as differentiated the flavor differences between grain cultivars. The developed model can be applied for the flavor evaluation of multiple grain types and can help researchers understand the flavor differences among grain cultivars. As a result, such knowledge will help to facilitate the selection of suitable products with favorable characteristics for specific applications as well as for selective breeding purposes.

## 1. Introduction

Grain is an important part of the human diet. According to the current report from the USDA, the total world consumption of wheat, rice/millet, and corn is 742.9 MMT (million metric ton), 475.5 MMT, and 1063.2.9 MMT, respectively [1]. The intake of whole grains has recently gained considerable attention due to its potential health benefits. For instance, the phytochemicals and antioxidants in whole grains have positive impacts on human health. According to Liu [2], whole grains are rich sources of phytochemicals (e.g., phenolic acids, 3-deoxyanthocyanidins, and tannins), vitamins (e.g., Thiamine and B6), minerals (e.g., copper, magnesium, and phosphorus), inulin, resistant starch, and beta-glucans, a class of soluble fibers that the FDA has proved to lower blood cholesterol levels. Many studies also have reported an association between the intake of whole grains and a reduction in the risk of developing cardiovascular disease, cancer, respiratory diseases, infectious diseases, diabetes, as well as non-cancer and non-cardiovascular diseases [3,4,5].

Each type of grain has a gene pool with a large amount of genetic variability. The huge genetic variability is responsible for the numerous varieties that are vital for specific applications or cultivation purposes. Bhattacharya [6] defines cultivars as plants that are selected based on desirable characteristics that can be maintained for propagation purposes. The variability in genetic, nutritional, physical characteristics, and growing conditions can yield differences in flavor profiles of grains among cultivars. Understanding the flavor profiles of different cultivars and varieties can help scientists, researchers, and industry professionals to select and develop favorable food solutions [7]. Such understanding also enables selective breeding efforts and supports product development, storage, and cultivation purposes. There have been various studies conducted to investigate flavor differences among products of different cultivars, as well as how storage conditions and importation might have different impacts on sensory characteristics among cultivars. In particular, multiple studies investigated the flavor differences of fruit cultivars, such as apples [8], nectarines [9], blueberries [10], cherries [11], mangoes [12,13], olives and olive oil [14,15], rose apples [7], and sweet tamarind [16]. Moreover, other studies were conducted on the topic of multiple types of vegetables, including tomatoes [17], perilla frutescens [18], potatoes [19], carrots [20], capsicum [21], and pumpkins [22]. Other authors have studied species of fish [23]. In addition, Koehler and others [24] examined 36 cultivars representing dry beans and determined the cultivar effects on nutrient composition, protein quality, and sensory properties.

Unlike the extensive work that has been conducted on fruits and vegetables to identify the effects of cultivars on the sensory properties of products, studies of this sort on grain varieties remain limited. This is surprising given that consumers routinely indicate that “taste” or sensory properties are the most important aspect of when choosing food [25,26,27]. Some studies have been conducted to investigate the sensory aspects of rice [28,29] and wheat [30,31,32] varieties. In addition, some studies have compared grains in finished products [33,34]. However, there have not been many studies conducted for most other grains and cereals, such as barley, quinoa, buckwheat, millet, sorghum, or rye, where differences in flavor among cultivars clearly exists. The number of studies conducted for those grain types is rare, and, if conducted, these studies are limited either in application methods or in the selection of samples. For example, Kobue-Lekalake and others [35] studied the effects of the phenolic compounds in sorghum grains on its bitterness, astringency, and other sensory properties, but they only looked at a single cooked grain application for six African-grown sorghums.

Studies on genetically diverse varieties are needed in order to capture the full spectrum of flavor profiles within a grain crop. Because the flavor properties of grain varieties can be important to the sensory properties of finished food products, a method for studying those variations in model systems is needed. Such model systems that are representative of various finished food systems do not currently exist for grains. Therefore, the aim of this study is to develop a model system(s) that could be used to conduct further research to characterize the flavor profile of grain varieties. This can support differentiation among cultivars in both grain form and in finished products. The study was conducted using sorghum grain as a model. Sorghum was chosen because there are enough differences and variations among the commercially available sorghum samples that allow sorghum to effectively serve as a model for the scope of this project.

## 2. Materials and Methods

Grains were evaluated in both their whole grain and milled forms. This project was approved by the Kansas State University Institutional Review Board for Human Subjects as “exempt”. Different cooking methods and procedures were applied to determine their effectiveness in evaluating the range of flavors. After a careful selection and consideration of various types of cooking procedures, five cooking methods were chosen to study further: cooked grains, porridge, cookies, muffins, and extruded puffed snacks. Those products were selected because they represent liquid preparation, baked goods that are widely made from various grains (that are not completely dependent on gluten formation), and a widely consumed snack food type.

### 2.1. Cooking Methods

The cooking methods were determined based on available literature on common applications and the cooking methods of grains as well as whether or not the applications were initially applied for the flavor characterization of grains. The cooked grain method was chosen because cooked grain is a common application for many grains; in addition, many studies that investigated flavor differences among different cultivars of rice utilized this procedure [28,29]. Kobue-Lekalake, Taylor, and de Kock [35] also characterized flavor differences among sorghum grains based on the cooked grains method. Porridge is another popular application of grains and thus was chosen as an application for the investigation of this study. In particular, cooked porridges were used to conduct tolerance testing of cooking procedures for fortified blended foods made from sorghum [36]. Various studies suggest that cookies and muffins can serve as effective models to characterize the flavors of grains. For example, many studies on sensory properties among wheat varieties use a cookie application to investigate the flavor profile differences among cultivars [30,31]. In addition, muffins also were used as tools to study nutritional composition, texture, appearances, and sensory acceptance among barley cultivars [37]. Finally, an extrusion application was chosen for the study in the form of puffed snacks to represent the extruded snack applications of grain varieties.

### 2.2. Samples

Nine commercially available sorghum samples were chosen for this study. All samples are available for food application purposes in the US market. Although nine samples is a relatively small number compared to the large number of cultivars available, they represent a broad range of types of sorghum samples from light to dark in color and standard vs. waxy vs. popping types. The number of samples is also larger than that used for other recent studies to compare cultivars of various grains or vegetables [32,35,38]. The samples were provided by Nu-Life Market (Scott City, Kansas) and Archer Daniels Midland (ADM, Chicago, IL, USA). Eight samples were provided by Nu-Life Market and one sample was provided by ADM in whole grain and milled flour forms. Of the eight sorghum varieties provided from Nu-Life, four were tan cultivars, three were burgundy, and one was a black sorghum. These samples are different from each other not only in color but they also have different genetic makeup and functional qualities. For instance, these varieties were classified by their tannin content, as waxy or non-waxy, and by functionality, such as specified for baking, specified for extrusion, and specified for popping. The specifications and information regarding the samples were provided by each company. The sample from ADM is tan in color and is specified for general application. Samples were stored at room temperature until used. General information regarding samples is provided in Table 1.

### 2.3. Sample Preparation and Serving

Cooked whole grain: To prepare the cooked grain samples, sorghum grains were cooked in deionized water using a ratio of one part whole grain to three parts treated water (reverse osmosis, carbon filtered). The detailed procedure includes measuring the appropriate quantity of grains, the rinsing of sorghum grains, bringing the approximate amount of grains and water to the boil, reducing the heat down to medium-low, and cooking for approximately 45 min (Table 2). The samples were occasionally stirred and additional water was added if necessary to maintain sufficient liquid for cooking. Any excess water was strained, and samples were served to the panelists while still warm in 120-ml Styrofoam cups covered with lids and labeled with 3-digits codes.

Porridge: To prepare the porridge samples, a ratio of one part flour to nine parts of treated water was used. To prevent the samples from clumping, the flour was measured in a large bowl and mixed with four parts of water to disperse the flour. This mixture was placed in a saucepan and the remaining five parts water was added. The sample was brought to a boil before simmering on low heat for 8 min while continuously stirring with a wooden spoon. Samples were served to each panelist approximately 10 min after cooking, while still warm, in a 120-ml Styrofoam cup covered with a lid and labeled with 3-digits codes. 

Cookies: Cookie samples were prepared according to the recipe and procedure listed in Table 3. The cookie recipe was developed particularly for this project. Initially, the AACC’s regular cookie recipe (Baking Quality Cookie Flour-Micro Wire-cut Formulation, AACC International. Method 10-54.01) was used. However, based on panelist feedback, it was hard to characterize the flavor profile of samples because the samples were quite sweet and the leavening was too strong, overwhelming the grain’s characteristic flavor notes. Therefore, the recipe was modified with reduced sugar and baking soda to provide cookies that still had a typical texture and flavor of a cookie, but successfully highlighted the different flavor characteristics of sorghum grains in a baked grain product. After the cookie samples were baked, they were allowed to cool for around 1 hour, vacuum sealed in bags (Food Saver, Newell Brands, NJ) and kept at room temperature until serving. The samples were opened 1 hour before serving and were presented in 3.25-oz. cups with lids and labeled with 3-digit binding codes. Each panelist received 1 cookie and additional samples were served to panelists as requested.

Mufffins: For muffin samples, a standard plain muffin recipe and procedure were used (Table 4). All samples were prepared approximately 1.5 h before serving. After baking, the samples were allowed to cool for about 30 min, cut into quarters, and were served in 3.25-oz. plastic cups with lids, coded with 3-digit codes. Each panelist received one muffin and additional samples were served upon request.

Extruded Puffs: For the extruded puffs samples, sorghum flours (moisture content from 8%–10%) were pre-mixed in a Hobart planetary mixer with water to reach a moisture content of 15%–16%. The pre-moistened flours were transferred into sealed plastic bags (Ziploc, S.C. Johnson & Son, WI) and were left to hydrate overnight in a refrigerator. The sorghum puffs samples were extruded using a lab scale co-rotating twin screw extruder (American Leistritz, Somerville, NJ, USA) with a circular die with a 3.2 mm diameter. The screw speed was kept constant at 400 rpm and the extruded products were cut into approximately 20–25 cm strands using a stainless-steel kitchen knife. The products were dried in the oven at 60 ℃ for approximately 2 h. After the drying step, the products were stored in zip-top plastic bags at room temperature. All extruded samples were served within one week after being processed. The samples were served to panelists in a 100-mL (3.25 oz.) cup labeled with 3-digit binding codes.

### 2.4. Panelists

Six highly trained panelists from the Center for Sensory and Consumer Behavior, Kansas State University (Manhattan, Kansas), participated in the study. All of the panelists completed 120 h of panel training for descriptive analysis on a variety of food and non-food products. Specifically, the panelists received training and practice in attribute identification, terminology development, and intensity scoring. Moreover, the panelists have extensive experience on descriptive analysis with more than 1000 h of testing and evaluation of a variety of food products and received orientation specific to this project. Such numbers of highly trained panelists have been shown to be able to discriminate among samples better than larger panels of less trained panelists [39,40,41]. Similar panels have been used for other recent studies [42,43,44].

### 2.5. Evaluation Procedure

Ten 1.5-hour sessions were held for term generation and the assessment of all samples prepared from the 5 cooking applications used to develop the model system. Panelists were served 4–5 samples per session and were asked to provide terms to describe the flavors associated with each product. The panelists also were asked to provide feedback regarding how effective the products were in highlighting the flavor of the base grain and any issues that needed to be addressed in the samples that may have caused problems with the samples. Modifications to sample preparation were made based on panelist feedback. For example, panelists commented on texture inconsistencies in some samples that affected the flavor characterization of samples or levels of added ingredients that overpowered base grain flavors. This process continued until consensus was reached among panelists. The panelists evaluated samples using the consensus method, because it is convenient for any necessary modifications or adjustments to be made during the process [45]. The results were gathered and comparisons on the effectiveness of each cooking application were made to identify their effectiveness in characterizing the grain flavors.

## 3. Results and Discussion

Table 5 summarizes results from the term generation process for all five cooking applications: cooked grains, porridge, cookies, muffins, and extruded puffed snacks. The cookie application has the highest number of generated terms with a total of 37 terms identified from the nine sorghum cookie samples. Some of the terms include acrid, bran, brown, leather, burnt, buttery, beany, corn-like, fruity, heated oil, leavening, metallic, salty, toasted, wheat-like, and woody. For the cooked grains procedure, there were 27 terms generated, such as astringent, beany, bitter, bran, cardboard, earthy, nutty, petroleum, sour, wheat-like, and woody for the sorghum samples. The panel generated 23 descriptive terms to describe the flavor of nine sorghum porridge samples, while the number of terms generated from the muffin samples was 21. There were only 18 descriptive flavor attributes identified by panelists based on the nine extruded puffed snack samples. Panelists indicated that all the attributes identified in each model system differentiated among one or more samples in each model system. However, they did not score each attribute intensity because of the sheer volume of samples produced in this study.

The panelists’ feedback included that it was best to identify and characterize the flavor characteristics of sorghum varieties based on the samples from at least two of the cooking applications. The panelists noted that two methods provided cooked samples without flavors associated with browning (cooked grain, porridge) and that three methods provided some flavors associated with browning reactions (cookies, muffins, extruded puffs).

The cooked grain samples highlighted the distinct flavor attributes of the grains to provide clear differentiation among sample profiles. Even after considerable modification of the preparation procedure, the panelists described the porridge as having a texture that hindered the ability to focus on the flavor identification process. Specifically, the porridge samples were mouth coating and drying as well as gummy, adhesive, sometimes lumpy, and having an overall inconsistency in texture that sometimes left residue in the mouth that was hard to clean out. In addition, there were large differences in the functional characteristics among the various sorghum flour samples (cultivars) that resulted in overall textural inconsistency between porridge samples prepared from the same cooking procedure. Because no unique or distinct flavor terms other than those identified using the cooked whole grains procedure were found using the porridge samples (Table 5), they provided no benefit. Thus, as an application to characterize the flavor profiles of grain varieties, the porridge approach was eliminated.

The final cookie samples (made according to the recipe, Table 3) were “neutral” but emphasized the differences in each cultivar’s flavor. The cookie samples provided the largest number of flavor terms, including both based grain and “dry heat cooking” effects. All terms from the muffin samples were included in the list of terms for the cookie samples. Textural inconsistency between muffin samples also affected the flavor characterization process. Some muffin samples were gummier and others were gritty and had strong mouth drying effects. In addition, the intensity of the leavening attribute overwhelmed the distinct flavor notes of the grain. Reducing the amount of leavening and the lack of any gluten network in sorghum flour resulted in a poor muffin product. As a result, the muffin application was eliminated in the favor of the cookie model system.

The panelists noted that the extruded puffed snack samples were highly associated with many processing attributes, such as toasted and burnt. The extruded puff provided the fewest flavor notes and none of the attributes were unique to the extrusion application. Thus, the extrusion application was eliminated as a model system for testing grain flavors.

Together, the cookies and cooked grain applications cover all the identified attributes from all five cooking approaches and thus can serve as a representation to establish the flavor profile of grain varieties in both the whole grain form and in the finished product. The sorghum cookies made using the above recipe and procedure provide an effective model to facilitate the characterization of grain flavors.

This approach also was applied for flavor characterization of wheat varieties in a proprietary industry study and results confirmed the effectiveness of the model (data not shown).

There were some limitations associated with the study. The first limitation is tied to the limited number of sorghum varieties used for the project. The model was developed using nine commercially available sorghum samples, while literature has indicated much more genetic variations of sorghum grain. Additionally, more application of the model for other grain types, such as rice, barley, rye, quinoa, or millet, is necessary to confirm the generalized effectiveness of the model for grain flavor characterization. Finally, the descriptive sensory testing of samples made using this developed model system is necessary to provide a grain’s “flavor DNA” and to map products compared to each other to confirm the practicality of the model systems for characterizing flavors.

## 4. Conclusions

Understanding the flavor profiles of varieties and cultivars of grains to understand how they differ will help researchers, food producers, and those who cultivate grains. This research develops a model system to facilitate the characterization of flavor differences among grain cultivars. The model was developed using nine commercial sorghum samples that are different from each other in physical appearance, nutritional composition, as well as functional characteristics. A total of five cooking applications were examined and the results indicate that cooked grains and cookie applications had the most flavor attributes and may be an effective model for the flavor characterization of grains between different cultivars. This project developed a cookie recipe that creates consistent and neutral samples that successfully facilitate the identification of grain’s character notes. Further work to characterize a larger number of samples using these two methods, cookie and cooked grains, should be established to ensure that they successfully differentiate among cultivars in various grain types. These model systems can be used in future applications for demonstration purposes as well as in combination with descriptive analysis to characterize the flavor of grains and their differences among multiple cultivars.

## Figures and Tables

**Table 1:** Commercial available sorghum samples.

Company	Color	Specification
**Nu-Life**	Tan	Specified for Baking
		Specified for Extrusion
		Specified for popping of grain
		Waxy
	Burgundy	Tannin-free
		Tannin-containing
	Black	
**ADM**	Tan	Whole grain–general use

**Table 2:** Preparation of cooked grain samples.

Ingredients	Amount	Procedure
**Grain**	236 mL	1. Measure the amount of grains.
	(~190 gm)	2. Wash grain
		3. Drain and put in the pot.
**Water**	700 mL	4. Measure the amount of water, add in and bring the pot to boil.
		5. Lower the heat and let simmer for 45–50 min.
		6. Drains excess water and serve.

**Table 3:** Cookie recipe and procedure.

Ingredients	Amount Per 100 g Flour	Procedure
**Granulated White Sugar**	10	Measure shortening and corn syrup into a mixer bowl. Measure all dry ingredients in a separate bowl; mix all the dry ingredients together until incorporated. Place the mixing bowl into the mixer, turn on low speed and let mix for around 2 min. Slowly add the dry ingredients mix to the mixing bowl while mixing (small portion at a time) and scrape down in between. Turn to slightly higher speed and mix for 2–3 min until small lumps/crumbs are formed. The amount of water can be varied based on grain variety. Start with a lower amount and add more as needed. Slowly add water to the mixture (this step needs to be monitored carefully, add water enough to form the dough but do not go over or the dough will get too wet and hard to work with). Lower the mixer speed to low and let mix for 2 min. Form the dough into a ball. Roll out the dough to a thickness of 0.4–0.5 cm. Cut into 2 inches diameter cookies and bake at 205 °C for 11 min. Note because color of samples can vary with the variety of grain, a standard time was used for all samples.
**Instant Nonfat Dry Milk**	1	
**Salt**	1.25	
**Baking Soda**	0.5	
**Shortening**	40	
**Light Corn Syrup**	1.5	
**Water**	Varies (20 mL–40 mL)	
**Sorghum Flour**	100	
**Total**	176.5	

**Table 4:** Muffin’s recipe and baking procedure.

Muffin Recipe	Amount (g) for Approx. 6 Muffins	Procedure
**Sorghum Flour**	112	1. Preheat the oven to 185 °C.
**Granulated White sugar**	12	2. Grease two 6-cup muffin pans or line with paper cups.
**Double Acting Baking Powder**	5.4	3. In a medium-large bowl, stir together dry ingredients: the flour, sugar, baking powder and salt. In a separate bowl, mix all the wet ingredients: oil, egg beater and milk, together.
**Salt**	3	4. Make a well in the dry ingredients. Immediately add the mixed liquid ingredients, stir with a rubber spatula until well blended, do not over mix.
**Egg Beaters Original**	24	5. Spoon the batter into the prepared cups, filling around half full of the muffin tins.
**Milk 2%**	125	6. Bake until the tops of the muffins spring back when lightly pressed, about 20–25 min. Cool in the pans for at least 10 min before removing.
**Vegetable Oil**	21	7. Cut the muffin in half and serve in a 3.25-oz. cup.

**Table 5:** Generated sensory terms from 5 cooking applications.

Cooking Method	Terms Generated			
**Cooked Grains**	Astringent	Cooked	Floral	Sour
	Beany	Corn-like	Green	Starchy
	Bitter	Dark Green	Heated Oil	Sweet
	Bran	Dusty	Musty	Umami
	Brown	Earthy	Nutty	Wheat-like
	Buttery	Eggy	Petroleum	Woody
	Cardboard	Fermented	Raw	
**Porridge**	Animalic (leather) *	Cooked *!	Heated Oil *!	Starchy *!
	Astringent *!	Dark Green *!	Metallic *	Sweet *!
	Beany *!	Dusty *!	Musty *!	Umami *!
	Brown *!	Earthy *!	Nutty *!	Wheat-like *!
	Cardboard *!	Floral *!	Oil *	Woody *!
	Chalky *	Fruity *	Salty *	
**Cookies**	Acrid	Chalky	Fruity	Raw
	Animalic (leather)	Cooked	Green	Salty
	Astringent	Corn-like	Heated Oil	Sour
	Beany	Dark Green	Leavening	Starchy
	Bitter	Dusty	Metallic	Sweet
	Bran	Earthy	Musty	Toasted
	Brown	Eggy	Nutty	Umami
	Burnt	Fermented	Oil	Wheat-like
	Buttery	Floral	Petroleum	Woody
	Cardboard			
**Muffins**	Baked	Dusty *!	Leavening *	Sour *!
	Bran *!	Eggy *!	Metallic *	Starchy *!
	Brown *!	Floral *!	Musty *!	Sweet *!
	Cardboard *!	Fruity *	Nutty *!	Toasted *
	Doughy	Heated Oil *!	Salty *	Woody *!
**Extruded Puffed Snacks**	Animalic (leather) *	Cardboard *!	Nutty *!	Sweet *!
	Bitter *!	Dusty *!	Oil*	Toasted*
	Bran *!	Floral *!	Sour *!	Wheat-like *!
	Brown *!	Fruity *	Starchy *!	Woody *!
	Burnt*			

**Notes:** (*) Terms included in Cookies. (!) Terms included in Cooked Grains. (*!) Terms included in Cookies and Cooked Grains. Note: Panelists noted that each attribute differentiated among samples in that cooking method, but did not score each individual sample.
